# Absence of microbiome triggers extensive changes in the transcriptional profile of *Hermetia illucens* during larval ontogeny

**DOI:** 10.1038/s41598-023-29658-x

**Published:** 2023-02-10

**Authors:** Laurence Auger, Sidki Bouslama, Marie-Hélène Deschamps, Grant Vandenberg, Nicolas Derome

**Affiliations:** 1 Département de Biologie, Université Laval, Quebec, QC Canada; 2 Institut de Biologie Intégrative et des Systèmes (IBIS), Département de Biologie, Université Laval, 1030 Avenue de la Médecine, G1V 0A6 Quebec, QC Canada; 3 Département des Sciences Animales, Université Laval, Quebec, QC Canada

**Keywords:** Gene expression, Entomology

## Abstract

Black soldier fly larvae (BSF, *Hermetia illucens*) have gained much attention for their industrial use as biowaste recyclers and as a new source of animal proteins. The functional effect that microbiota has on insect health and growth performance remains largely unknown. This study clarifies the role of microbiota in BSF ontogeny by investigating the differential genomic expression of BSF larvae in axenic conditions (i.e., germfree) relative to non-axenic (conventional) conditions. We used RNA-seq to measure differentially expressed transcripts between axenic and conventional condition using DESeq2 at day 4, 12 and 20 post-hatching. Gene expression was significantly up or down-regulated for 2476 transcripts mapped in gene ontology functions, and axenic larvae exhibited higher rate of down-regulated functions. Up-regulated microbiota-dependant transcriptional gene modules included the immune system, the lipid metabolism, and the nervous system. Expression profile showed a shift in late larvae (day 12 and 20), exposing a significant temporal effect on gene expression. These results provide the first evidence of host functional genes regulated by microbiota in the BSF larva, further demonstrating the importance of host-microbiota interactions on host ontogeny and health. These results open the door to optimization of zootechnical properties in alternative animal protein production, biowaste revalorization and recycling.

## Introduction

The study of microbial ecology in relation to animal production is widely investigated for its economic importance as interactions between hosts and their associated microorganisms are critical for the establishment of optimal biological processes including growth, development, and health^1^. As human population steadily grows and, consequentially, food demand, particularly for animal proteins, the black soldier fly (BSF) *Hermetia illucens* has gained increased importance as a prime candidate for industrial production of alternative animal protein, biowaste revalorization and recycling^2^. The investigation of host-microbiota interactions in *H. illucens* offers insight in how these biotic factors affect the insect’s physiology and can be used to optimize industrial rearing and bioconversion efficiency. Research to date on *H. illucens* microbiome has been focused on characterizing the taxonomic composition of the larval digestive tract’s bacterial and fungal communities, and the effect of diet on community assembly^3–8^. Still, the microbiota’s impact on life-history traits and biological processes of *H. illucens* is poorly understood. In this study, we developed a rearing method to produce axenic (i.e., germfree) *H. illucens* larva (BSFL) and used a transcriptomic approach to document the functional repertories that are regulated by the host’s microbiota.

Germfree animal models have been used to investigate the effect of microbiota on its host genomic expression by inferring that the genes differentially expressed in an axenic model are modulated by the microbiota. The time-dependent progression of gene expression profile is common to all organisms across evolution^9,10^. In germfree *Drosophila*, microbiota has a greater overall impact on the transcriptome in older flies. About 70% of the changes in gene expression conserved across species fail to occur in axenic flies, including the expected time-dependant decline in the expression of stress response genes and the increase of innate immune genes’ expression^11,12^. The germfree *Drosophila* also exhibits up-regulation of genes involved in metabolism, oxidative stress, lipid metabolism, and immune response in presence of microbiota, while genes encoding factors involved in transporters are down-regulated^11^. The sustained expression of stress response genes in germfree insects may be caused by the well-known ability of endosymbionts to detoxify xenobiotics for their insect host^13^.

While current germfree models are informative, Insecta is the most diverse Class of eukaryotes, occupying all ecological niches and exhibiting broad differences in life-history traits. Therefore, knowledge from other fly species models may not simply be generalized to *H. illucens*. Insect species in different niches are not exposed to the same free-living microorganisms, hence they are expected to have a distinct microbiota composition, such as the microbiota dissimilarities observed between *Drosophila melanogaster* and *Aedes aegypti*^12,14,15^. Given the fundamental changes that the microbiota operates on its host, investigating the effect of host-microbiota interactions on the host is of great significance to understand the mechanisms behind *H. illucens’* sought-after abilities, such as its bioconversion rate and antimicrobials properties, and to guide rearing approaches and to optimize exploitation potential.

Here we report on how the presence or absence of a microbiota changes the transcriptome profile of the host during larval development (day 4, 12 and 20 post-hatching) by comparing relative abundance of transcripts expressed in larvae reared in axenic *vs* in non-axenic (referred to as conventional in this paper) conditions.

BSFL feed on organic waste that usually contains heavy bacterial loads including potential pathogens, which suggests *H. illucens’* immune system is very efficient at controlling infection. The larva encodes up to 50 putative antimicrobial peptides (AMPs)—a record equalled only by one other insect *Harmonia axyridis*, an invasive beetle—that can reduce the abundance of potential pathogens in their environment (i.e., *Salmonella* and *Escherichia coli*)^16–19^. Gut microbiota is usually adapted to the specific environment it colonizes, exploiting stable resources generated by the host and in turn, providing protection to the host by competition with invasive pathogenic microorganisms (i.e., colonization resistance)^20^. In *Drosophila melanogaster*, the taxonomic composition of the gut microbiota modulates the induction of innate immune gene products^21,22^. Therefore, the first objective was to test if the transcriptional activity of genes in immune system processes were lowered in the axenic BSFL compared to conventional BSFL.

The microbiota associated with BSFL in compost has an increased activity of metabolic functional groups associated with carbohydrate-active enzymes^23^. Starvation has also been identified as a factor that alters BSFL’s gut microbiome^24^. These studies highlight the potential link between the expression of metabolic genes in the BSFL and the gut microbiota. While many insects are selective feeder, the polyphagous BSFL must adapt to a wide range of food components and defences, sometimes in a single life cycle. This quick adaptation is often attributed to phenotypic plasticity^25^. However, the gut-associated microbiota has emerged as a flexible metabolic resource for the host, facilitating adaptation to new food-sources, known as metagenomic plasticity^26,27^. Therefore, the second objective was to test if the transcriptional activity of genes involved in carbohydrate metabolism is lowered in axenic BSFL compared to conventional BSFL.

Microbiota also contributes to host processes like neurophysiology and behaviour^28^. Like most insects, *H. illucens* interact with their environment in great part through olfaction. In adult *H. illucens*, bacteria associated with deposited eggs attract oviposition by other conspecifics^29^. However, microbiota-brain axis during ontogeny has not been investigated to date. We observed that BSFL tended to leave their growth substrate (Gainesville) when exposed to a more odorant one (poultry hatchery waste) (unpublished work). This suggests olfaction also regulates behaviour in larval stage and may be associated with microbiota. Therefore, our third objective was to test if transcription of genes related to development, in particular nervous system and olfaction related genes, are downregulated in axenic larvae compared to conventional larvae.

Microorganisms have the metabolic ability to recycle toxic components in the environment into bioavailable molecules for multicellular organisms^30^. This detoxifying ability is exploited by insect hosts to protect themselves from secondary defense metabolites and xenobiotics^31^. Generalist insects such as BSFL often switch food source and are therefore prone to encounter a wider array of dangerous components than specialist feeders. Gut-associated bacteria rapidly adapt to the presence of xenobiotics in the environment by horizontal gene transfer of detoxifying genes from environmental bacteria or recruitment of new bacteria harboring adaptative genes^32^. Therefore, our fourth objective was to test the extent of the difference in transcriptional activity of genes associated with xenobiotic remediation and oxidative stress between axenic and conventional larvae.

## Methods

We measured the differential expression of transcripts in larvae reared in axenic versus in conventional condition at day 4, 12 and 20 post-hatching. The metabolic activity affected by the absence of microbiota in the host was investigated through functional annotation of the DETs.

### Production of sterile larvae in axenic condition

Eggs used in experiments were obtained from the black soldier fly colony maintained in *LAboratoire de Recherche en Sciences Aquatiques* (LARSA) at Université Laval (Québec, Canada). Flies are inbred to produce each new generation, producing a homogenic population.

Two experimental groups were compared: conventional (with microbiota) and axenic (without microbiota). Each group was carried out with 6 experimental replicates. For each replicate, 0.05 g of pooled egg clutches were transferred into a sterile cell strainer (SG-70ICS, MIDSCI) for sterile or control treatment.

Axenic eggs were produced using an adapted version of the protocol for the sterilisation of *Drosophila* larvae^33^. Eggs were submerged successively into 2.5% active hydrochloric acid, then (further steps under vertical laminar flow cabinet) into 70% EtOH with continuous shaking and then rinsed in 2 successive Phosphate-buffered saline baths (PBS, 137 mM NaCl, 2.7 mM KCl, 10 mM Na_2_HPO_4_, 1.8 mM KH_2_PO_4_, pH 7.0), each treatment was 3 min. After treatment, each egg pool was transferred until hatching on a sterile vented cell culture flask (0.3 μm filter; Denville^®^) with 50 mL agar culture media (pH 7.0 ± 0.2) composed of Brain–Heart Infusion (BHI) with added 10% Yeast extract Peptone Dextrose. Eggs were observed every 12 h until hatching; upon hatching 150 neonates were transferred into new flasks (n = 5) with sterile BHI medium for the experiment.

Eggs for conventional condition followed the same manipulations as axenic eggs except that bath solutions were replaced by PBS 1X.

For both conditions, larvae were reared on sterile growth media (BHI) in flasks and incubated for 20 days at 28 ± 1 °C, 70% relative humidity and 12 h:12 h photoperiodic cycle (VWR^®^ B.O.D Refrigerated Peltier Incubator, VRI3P 89,510-738).

### Verification of sterility

To verify that the larvae reared in axenic conditions were truly sterile, we used traditional microbiological methods and molecular test. For the traditional approach, we sampled from each flask 3 larvae that were crushed with a sterile pestle in 600 μl of BHI liquid culture. Immediately after sampling and 7 days later, 100 μl of the culture was inoculated on a BHI agar culture media (kept 7 days at 28 °C in aerobic conditions)^34^. This test was repeated for each sampling time, using conventional larvae as positive control. Sterility was constated when no growth (bacterial or fungal) was observed for axenic condition and growth was observed in control; if any growth was found on the culture media, the corresponding flask was discarded. Since this traditional method is limited to culturable microorganisms, we also proceeded with a molecular test to detect bacterial contamination by PCR amplification of the 16S V3-V4 rRNA gene region (Primers forward: 5′—ACACTCTTTCCCTACACGACGCTCTTCCGATCTCCTACGGGRSGCAGCAG—3′ and reverse 5′—ACACTCTTTCCCTACACGACGCTCTTCCGATCTGACTACHVGGGTATCTAATCC—3′)^34^. Bacterial DNA was isolated by the salt-extraction method described by Aljanabi and Martinez^35^. PCR started 2 min at 98 °C, followed by 30 cycles of 10 s at 98 °C, then 30 s at 60 °C and 30 s at 72 °C, with a final elongation of 2 min at 72 °C. The molecular test was done at day 4 and at day 20 using ~ 50 mg of larvae, the extracted DNA from each replicate was pooled together for the same condition for gel electrophoresis (2% agarose), results presented in Fig. 7. Sterility was confirmed when no band (or a very faint band, expected to be mitochondria DNA) was visible for the axenic condition while a band was visible at around 465 pb for positive control (bacterial culture) and conventional larvae^36^. Axenic replicate with visible band were discarded.

### Sampling process

At day 4, 12 and 20 post-hatching (hatching = day 1), a sample of 6 pooled larvae was taken from each flask. Sample were flash-frozen (liquid nitrogen) before storing at − 80 °C until RNA extraction. At each sampling, 10 more larvae were sampled from each flask to measure total length using a digital caliper (live larvae were put on a petri dish resting on ice for measurement).

### RNA extraction and sequencing

Total RNA of pooled larvae was extracted using TRIZol reagent (Invitrogen, Life Technologies) according to the manufacturer’s instructions with DNase I treatment to remove genomic DNA contamination. Quality of RNA was assessed with an Agilent 2100 Bioanalyzer and concentration was measured on a NanoDrop ND-2000 Spectrophotometer. Library preparation of poly(A)-enriched RNA (NEBNext^®^ Ultra™ II Directional RNA Library) and RNA-Seq were done by CES Génome Québec (Montréal, Québec, Canada) on an Illumina NovaSeq 6000 platform. Four samples were discarded because of poor RNA quality: One replicate from the conventional condition for each time point and one replicate from the axenic condition for day 12. Therefore, all following statistical tests are done with 5 replicates by condition by time.

### DETs functional annotation

Quality RNA-seq output reads were selected using *Trimmomatic* with Phred-equivalent scores < 20^37^. Transcripts assembly was done with Trinity (https://github.com/trinityrnaseq/trinityrnaseq/wiki) and read count was estimated with RSEM^38^. Read counts were normalized and measured for differential expression with DESeq2 using a pair-wise comparison between both conditions for each individual time point^39^. Differentially expressed transcripts (DETs) were filtered with *alpha* threshold 0.01 and an effect size threshold (log fold change, LFC > 1) to ensure no confounding expression levels affected the analysis of biological pathways.

Transcript isoforms produced by Trinity were processed to detect open reading frames (ORFs) and predict resulting proteins. All orphan transcripts were translated into amino acid sequences to predict annotation by BLASTP against the UniProt database, as well as BLAST against the Pfam database (http://pfam.xfam.org/).

Functional annotation was made by BLASTX on transcript sequence, and BLASTP on predicted proteins from ORFs, against the Uniport-Swissprot database^40^ to retrieve KEGG (Kyoto Encyclopedia of Genes and Genomes^41^) and GO (Genome Ontology) annotation from resulting matches. Gene ontology annotation was done on the DETs by assigning GO terms using “Quick Go” (https://www.ebi.ac.uk/QuickGO/).

Gene set enrichment analysis (GSEA) of GO terms was done by performing a hypergeometric test to investigate biological functions and pathways associated with DETs^42^. This allows the detection of DETs with a low individual effect but belonging to coordinated groups of expression profiles^43^. GO terms were filtered for significance threshold (*p*-adjusted value < 0.01, false discovery rate, FDR < 0.01) to conserve only relevant terms. GO terms were also compared between conditions at each time point to remove shared terms to focus our analysis on the biological difference between conditions.

Associated pathways were further examined with a list-based pathway enrichment analysis in KEGG database^41^ providing a dataset of transcript abundance for each pathway.

## Results

### Larvae exhibited dwarfed growth on culture media

**Figure 1:**
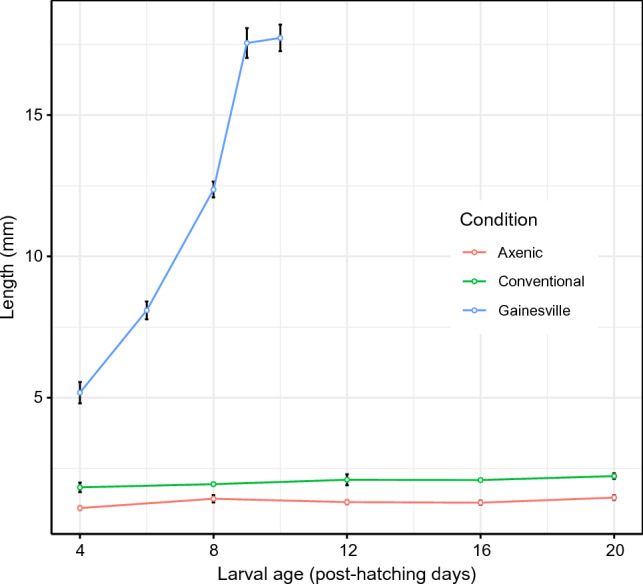
Length of larvae reared on Gainesville or BHI. Total larval (N = 10) length variation on a period of 20 days (day 1 = hatching), of larvae reared in axenic (red) and conventional (blue) conditions and fed with BHI culture media and larvae from parent colony (green) fed with Gainesville feed (27̊C, RH 70% and 12:12 light cycle).

Sterilised eggs successfully hatched on sterile culture media. Larvae in both conditions survived using only BHI culture medium as feed for up to 20 days (end of experiment). However, larvae in both conditions did not show the expected growth. Compared to parent colony (i.e., larvae reared on the reference Gainesville diet (50% bran, 20% corn, 30% alfalfa, 70% RH^44^ )) both conditions had significantly smaller larval length (*p*-value < 0.05, SE = 3.129) with no difference between experimental conditions (p-value > 0.1, conventional min = 1.8 mm, max = 2.2 mm, IC95 mean = 2.08, SE = 0.056; axenic min = 1.1 mm, max = 1.4 mm, IC95 mean = 1.34, SE = 0.04; Gainesville min = 5.1 mm, max = 17.7 mm, IC95 mean = 12.18, SE = 0.70) (Fig. 1).

### Expression profile is characterized by rearing condition

We found 150,214 transcripts overall with a differential expression in pair-wise comparison at each time point between both conditions. To ensure the biological significance of these results, they were filtered by an effect size threshold (log-fold change, LFC > 1), resulting in 1 792 transcripts with a differential expression of abundance (adjusted *p*-value < 0.01) between axenic and conventional larvae at day 4, 12 and 20.

**Figure 2:**
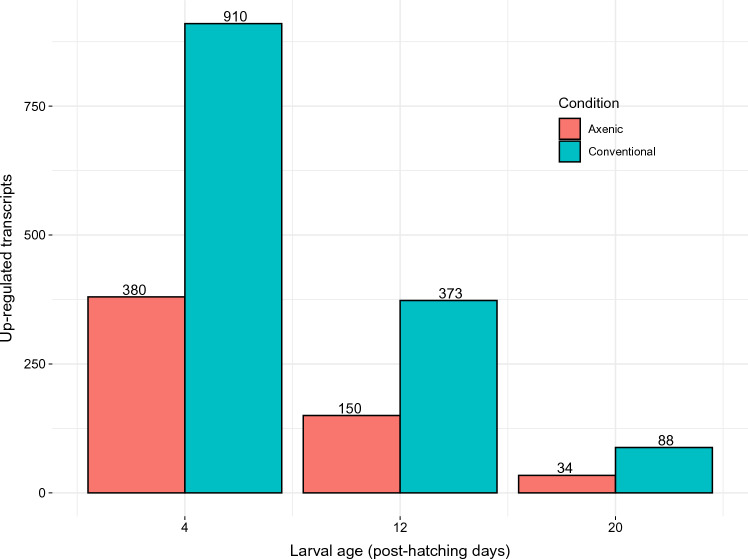
Differentially expressed transcripts upregulated for each condition. In red are the number of DETs upregulated for axenic and in blue for conventional larvae (N = 5 pools of 6 larvae/condition/time point) at the time points 4, 12 and 20 (day 1 = hatching).

Conventional larvae had consistently more DETs up-regulated than axenic larvae (Fig. 2). A decrease in the number of significant DETs was observed as time progressed. This was not caused by sequencing bias, as before filtering for significance we initially found 66 106, 103 412 and 115 826 transcripts DE at day 4, 12 and 20, respectively.

**Figure 3:**
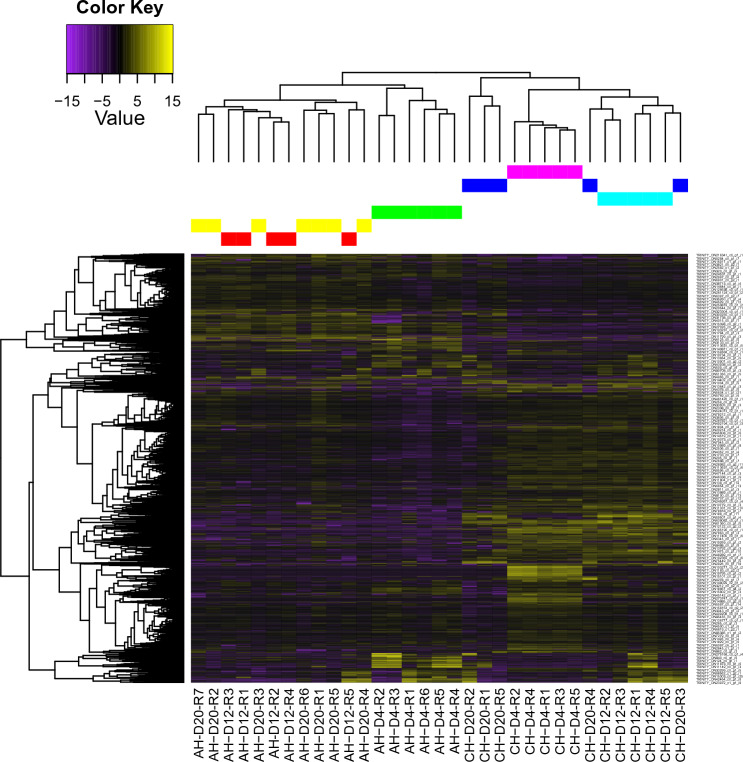
Heat map showing the DETs between axenic and conventional larvae. AH and CH refer respectively to axenic and conventional condition, for each time points (D4: day 4, D12: day 12 and D20: day 20), and the R indicates the replicate number. Transcripts with DE values of log2FC > 1 with adjusted *p*-value < 0.01 were clustered together based on expression pattern, as represented by the dendrogram at the top of the figure, each color coding for each combination of condition and time (yellow = Axenic day 20; red = Axenic day 12; green = Axenic day 4; dark blue = Conventional day 20, purple = Conventional day 4, blue = Conventional day 12). Presented values are for unique DETs arbitrarily named with Trinity (on the right). Measured phylogeny of transcripts is present on the left.

Cluster analysis of the expression profile patterns resulted in two major clusters; the major effect factor was the rearing condition (Fig. 3). Further sub-clustering was observed for day 4 in each condition, underlining the difference in expression profile in early larval development compared to later stages.

**Figure 4:**
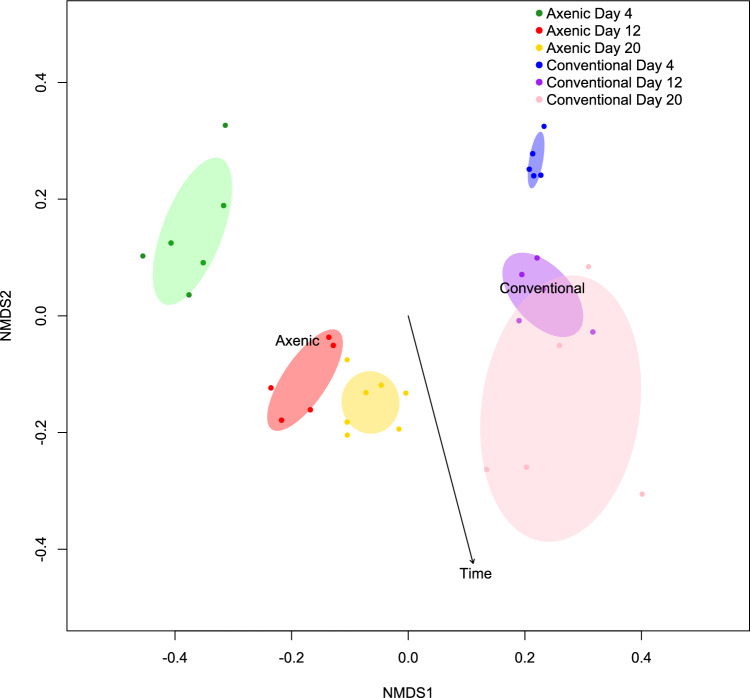
Similarity between expression profile of DETs in axenic and conventional larvae. Ordination based on samples transcripts by Jaccard test with non-parametric distances (N = 5). The test is fitted with time as numeric variable (confidence interval = 99% inside ovals).

To further explore sample transcriptome profiles, we did a Jaccard non-metric multidimensional scaling ordination analysis based on expression profile^45^. Results separated larvae into axenic and conventional condition distinct clusters, consistent with the previous differential analysis of gene expression (Fig. 4). Time was used as a fitted numeric variable. Axenic larvae had greater variation in their expression profile for early development (day 4) while later stages converged toward a more homogenous profile. Conventional larvae had the reverse tendency, growing towards a more heterogenous group in time.

### Functional analysis of DETs

The function of the up-regulated DETs was investigated by functional annotation with the public database UniProt Swiss prot using an e-value cut-off of 1e^−3^. We found matches for 886 (49.4%) of the DETs. Of the matched transcripts, 590 (66.6%) matched best to genes from *Neoptera* infraclass of the *Insecta* class. No transcript annotated to fungal genes with BLASTX.

**Figure 5:**
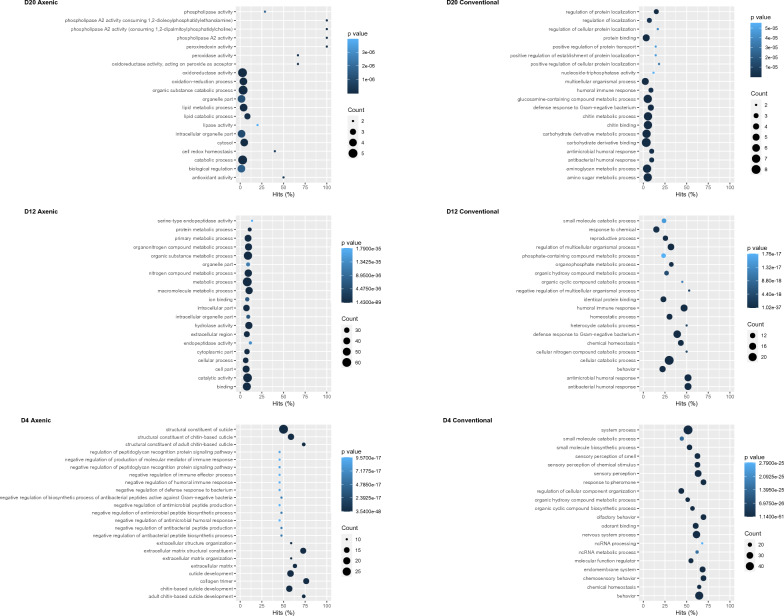
Top 20 terms in the GO enrichment analysis unique to each condition. GO terms that were unmatched (NA) are excluded. The 20 GO terms unique to a condition with the largest DETs ratios are plotted in order of the ratio. The size of the dots is representative of the number of transcripts in the significant up-regulated DETs list (p-adjusted value < 0.01, LFC > 1) associated with the GO term, while the coloration represents the corresponding p-adjusted value of the GO term (FDR < 0.01). The hits axe normalises the number of DETs in the category in relation to the total of genes implicated in this category.

The 20 most enriched ontologized terms following filtrations are presented in Fig. 5. Day 12 axenic larvae showed a preponderance of transcripts associated with the negative regulation of immune system processes. At day 20, GO terms related to catabolic processes, oxidoreduction and lipid process, as well as oxidative stress were enriched significantly in the axenic condition compared to the conventional condition.

Day 4 conventional larvae had a contrasting tendency with multiple enriched transcripts that included the term “*positive regulation”* (signaling the activation or increase in frequency of related process), for diverse biological processes including *positive regulation of immune system*. Furthermore, olfactive processes, *ketone biosynthetic process* and *ecdysteroid metabolic process* were enriched. *Nervous system process* was amongst the most significantly enriched GO terms in conventional larvae. The 12-days-old larvae had multiple enriched catabolic and metabolic processes, along with *behaviour* and *locomotion*. At day 20, *lysozyme activity* and terms pertaining to biosynthesis and degradation of glycogen were enriched. Interestingly, the *peptidoglycan muralytic activity* was also enriched; muropeptides are involved in symbiotic associations, microbial interactions, and pathogenesis in animals and plants^46^.

### Persistently up-regulated transcripts

Some of the DETs were found to have sustained up-regulation in a specific condition at all times (day 4, 12, and 20); annotated DETs persistently expressed in larvae in each condition are presented in Table 1. Axenic larvae exhibited continuous over-expression of digestives enzymes whereas conventional larvae showed consistently up-regulated genes related to transport, immune system, and structure.

**Table 1:** Annotated DETs persistently upregulated.

Condition	DET ID	Accession ID	BLASTX UniProt description	BLASTP UniProt description
Axenic	TRINITY_DN232849_c0_g2_i1	Q90629	Trypsin II-P29	–
	TRINITY_DN15398_c0_g2_i1	P54629	Trypsin eta	–
	TRINITY_DN17373_c0_g2_i2	P35032	Trypsin-2	–
	TRINITY_DN15398_c0_g2_i3	P54629	Trypsin eta	–
	TRINITY_DN1426_c0_g1_i10	P40313	NA	Chymotrypsin-like protease CTRL-1
	TRINITY_DN217518_c0_g1_i1	Q75VN3	Translationally-controlled tumor protein homolog	–
	TRINITY_DN20544_c0_g1_i11	Q8INK6	Peptidoglycan-recognition protein LB	–
Conventional	TRINITY_DN2418_c0_g1_i1		NA	Transcription activator MBF2
	TRINITY_DN143507_c0_g1_i1	O45599	Chitin-binding domain protein cbd-1	–
	TRINITY_DN88238_c0_g1_i2	Q3BAI2	Uncharacterized protein ORF91	–
	TRINITY_DN6492_c0_g1_i3	P35458	Dynactin subunit 1	–
	TRINITY_DN2302_c0_g1_i1	P19967	Cytochrome b5-related protein	–
	TRINITY_DN685_c1_g1_i1	Q3BAI2	Uncharacterized protein ORF91	–
	TRINITY_DN2104_c0_g1_i14	P91793	Defensin-A	–
	TRINITY_DN8022_c0_g1_i4	P18684	Diptericin-D	–
	TRINITY_DN130490_c0_g1_i5	P82174	Lysozyme	–
	TRINITY_DN49180_c0_g3_i1	Q3BAI2	Uncharacterized protein ORF91	–
	TRINITY_DN19867_c0_g1_i1	Q91XA9	Acidic mammalian chitinase	–
	TRINITY_DN3133_c2_g1_i5	Q17040	Protein G12	–

### Biological pathways enrichment

**Figure 6:**
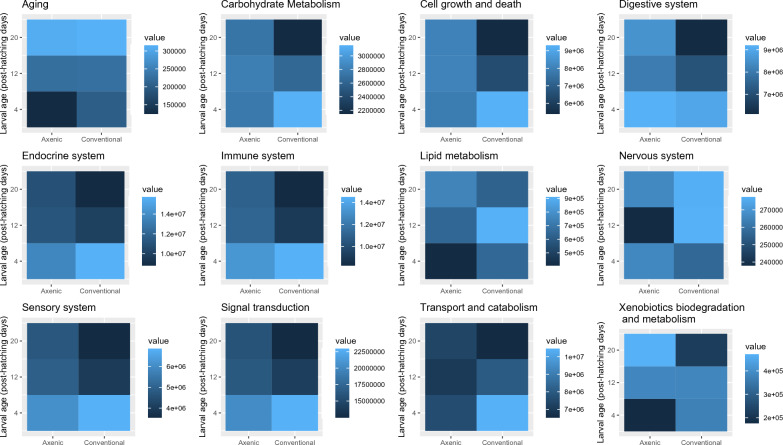
Enriched KEGG pathways in larvae reared in axenic and conventional condition. The value represents the total abundance of DETs associated with the specific pathway for the condition at each time point.

Pathways enrichment for the 1792 DETs focussed on 12 pathways distributed in four KEGG categories, namely Metabolism, Environmental Information Processing, Cell Processes, and Organismal Systems (Fig. 6).

## Discussion

Host-microbiota interactions must be properly addressed when trying to understand and optimize animal rearing for industrial purposes. Indeed, microbiota works as an added genomic arsenal for the host that can affect directly or indirectly its own biological functions and processes^26,47–49^. Previous studies have characterized the taxonomic composition of bacterial and fungal communities associated with BSFL, highlighting variations correlating with abiotic factors^5,6,19^. However, these metagenomic and metabarcoding approaches have a limited capacity to characterize and quantify the effect of microbiota on host functional activity. Therefore, using a functional investigation of the host’s active biological processes through a transcriptomic approach allowed the accurate identification of host-microbiota interactions and highlighted the host functions that are regulated by microbiota activity. Here, we delved into how the microbiota’s presence (conventional condition) or absence (axenic condition) changes the transcriptome expression profile of the BSFL during larval ontogeny. This enabled us to gain insight into the most affected metabolic processes and biological pathways with subsequent functional annotation (UniProt), GO enrichment, and KEGG pathways enrichment. This study offers new insight into the host-microbiota interactions affecting BSFL ontogeny.

This transcriptomics study opens the way to metabolic studies, as it offers insight for more targeted studies. We report predicted pathways affected by the microbiota, however transcriptional activity gives little information on protein activity, which is the most relevant to fitness, and cannot substitute for detailed functional analysis^50^. In the future, metabolic studies are needed to carefully characterise the activity of metabolic pathways under axenic conditions for the BSF.

### Microbiota has a major impact on early larvae expression profile

While no length difference was measured between both experimental conditions, BHI reared larvae were significantly smaller than the parent colony reared on Gainesville substrate (Fig. 1). Since the feed source was the only difference in abiotic factors between the conditions of this experiment and the parent colony, we conclude the nutritional needs of larvae reared on BHI medium weren’t met. It is hypothesised that most of the larva microbiota is recruited from the environment, therefore we suspect the microbiota associated with the egg, which was the only microorganisms available in the conventional condition, do not reflect the natural microbiota of the larva and could have contributed to the dwarfed growth of the conventional condition compared to the parent colony. As both experimental conditions were under the same starvation stress, the differences in genomic expression are nonetheless relevant, the purpose of this study being to identify transcriptional differences between axenic and conventional larvae. Further study should be done to find a sterilisable substrate able to meet the nutritional needs of the larvae, to eliminate the starvation stress potential impact on the differences observed between both conditions. We found the standard feed used in BSFLrearing (Gainesville feed) to be very difficult to sterilise^44^. The gamma radiation method did not give desired results, and repeated autoclaving (121 °C for 30 min) changed the physicals property of the feed, making it unusable^51^.

The greater number of DETs up-regulated in conventional larvae at all time points (Fig. 2) indicates the microbiota-dependent co-expression of multiple genes in *H. illucens.* Conventional larvae had respectively 63%, 69% and 56% of upregulated DETs at days 4, 12 and 20.

The general dampened transcriptome expression observed in axenic larvae for multiple pathways (Fig. 6) suggests that the absence of microbiota results in widespread down-regulation of most pathways in early life stages. This generalized down-regulation effect is substantiated by studies on *Drosophila melanogaster* and other germfree animal models that unveiled the far-reaching effect of microbiota activity on host expression of genes involved in metabolism, gut structure, immune response, and the nervous system^11,52,53^.

Pathway enrichment analysis also indicates that microbiota induces a more targeted effect, some specific transcriptional modules being only activated or strongly expressed in presence of microbiota. The up-regulated DETs found in axenic larvae may correspond to genes inhibited by microbiota activity.

The absence of microbiota had the most pervasive impact on transcriptome profile in early larvae (day 4). In both experimental groups, the expression profile of early-stage larvae was independently clustered whereas it converged at later time points (Figs. 2, 3). Therefore, time was an important factor of transcript abundance, leading towards a more similar, condition dependent, expression profile in later ontology. However, the expression profile in axenic larvae had more heteroscedasticity in early larvae (day 4) than later stages, while conventional larvae presented inversely (Fig. 4). This suggest that microbiota plays an important role in early development and may help specializing early larvae expression profiles, a congruent result with the recognized concept of a critical window in early life during which a healthy microbiota is essential to the normal development of the host, independently of organism type^9,10,54^.

Axenic larvae exhibited an overall decreased metabolism compared to conventional larvae. This was expected as the microbiota is recognized for communicating with its host through the secretion of metabolic by-products, mainly short chain fatty acids that are crucial for normal metabolic functions^55,56^. Looking at KEGG pathways enrichment suggests that the absence of microbiota triggered a diapause state, a genetically programmed developmental arrest common in insects used to survive temporary adverse environmental conditions. This diapause state may be the result of an anti-stress physiological response to nutritional imbalance in sterilised BHI^57^. Axenic conditions may have induced the diapause state earlier. For instance, conventional larvae showed enrichment of *ecdysteroid biosynthetic process* GO at day 4 and 12. In *Bombyx mori*, the secretory rate of ecdysteroids is drastically reduced during diapause^58^. Microbiota activity likely prevented or delayed conventional larvae from entering diapause by providing enough accessible nutrients to the host. To date, the extent of the regulation of diapause biological processes by host-microbiota interactions in this species is unclear. In *Nasonia vitripennis* (a small parasitoid wasp), microbiota has an important role in the host’s nutrient allocation during diapause by maintaining glucose and glycerol levels^59^. The time-dependant down-regulation of pattern-recognition proteins in this model indicates a strong repression of the host’s immune system during diapause, which we observed in conventional BSFL and, at lesser degree, in the axenic conditions. Conventional BSFL also had increased expression of aerobic glycolysis (up-regulation of ectonucleoside triphosphate disphosphohydrolase) in early larval stage, contrary to the increased anaerobic glycolysis observed in several other dipteran models during diapause^60,61^. The diapause state induces major physiological and metabolic changes in the host. As microorganisms carry out metabolic processes for the host, it is expected that host-microbiota interactions play an important role in diapause processes^62^.

### Microbiota activates immune system processes in BSFL

Early (day 4) conventional larvae had enhanced immune transcriptomic activity of AMPs (i.e., cecropins, attracins, diptericins and defensins) as well as enrichment of the Toll signaling pathways. In *Drosophila,* the antimicrobial response to microorganisms is regulated by two major signaling pathways, immune deficiency (Imd) and Toll^63^. The enhanced expression of diptericins (Imd pathway) in our study shows a similar response in the BSFL to that of *Drosophila*, for which a basal level of expression of the Imd pathway is known to be induced by the microbiota^64^.

Some immune response activity was uniquely enhanced in axenic larvae, mainly peptidoglycan recognition proteins at day 12 and 20. Previous studies have found evidence that diet components can induce in *H. illucens* the expression of AMPs in a profile similar to bacterial-dependant immune response inhibitory activities, while endosymbiont microbiota can inhibit the host’s immune response to ensure its own survival^65^. This may explain why some immune responses were found uniquely in axenic larvae. This mechanism may be an evolutionary response to a dietary trigger; the antibacterial activity happened even in the absence of the targeted bacteria spectrum (or any bacteria), while the same response was inhibited in the presence of microbiota^19^. Immune system pathways showed higher enrichment at day four in conventional larvae, supporting previous results, but day 12 and 20 had higher enrichment in axenic larvae (Fig. 6). At day 12, axenic larvae had multiple enriched GO terms related to the regulation of immune processes and the negative regulation of immune processes (Fig. 5). This doesn’t correlate with an increase in immune processes. On the contrary, it suggests that the higher transcriptomic activity down-regulates immune processes. This is a crucial distinction that can be made when assessing the genomic expression through a combination of multiple tools such as differential expression annotation, functional enrichment, and pathway enrichment. This first discovery step opens the way and offers targets pathways for further research exploring the effect of the axenic condition at the protein level and better characterise how the immune process is modulated with the microbiota.

### Xenobitotic biodegradation and metabolism

The BSFL’s unusual ability to digest pesticides, mycotoxins and other xenobiotics raises questions on the role the microbiota plays in these biodegradation processes^66–69^.

We found, through pathway enrichment analysis, that while early conventional larvae had a higher expression rate of genes implicated in the xenobiotic biodegradation and metabolism, axenic larvae had the highest expression level in this pathway at day 20. Axenic larvae at day 12 had multiple up-regulated DETs encoding for cytochrome P450, which was recently found to be responsible for the metabolization of the mycotoxin aflatoxin B_1_, for which BSFL are known to have high tolerance and no metabolic accumulation^70,71^. Later axenic larval stages (day 12 and 20) also had up-regulation of the UDP-glucuronosyltransferase-2C1 (AC: P36514), a protein of major importance in elimination of potentially toxic xenobiotics and endogenous compounds, not found in conventional larval expression profile. This higher enrichment for the xenobiotic pathway could be a metabolic response to the accumulation of BSFL dejections in the substrate exacerbated by the absence of microorganisms able to recycle them. Early conventional larvae pathway enrichment combined with late over-expression in axenic larvae suggest there might be a synergy of metabolic processes in host-microbiota interactions, further studies are needed to explore the role of microbiota in BSFL bioremediation processes.

### Energy and digestive systems

**Figure 7:**
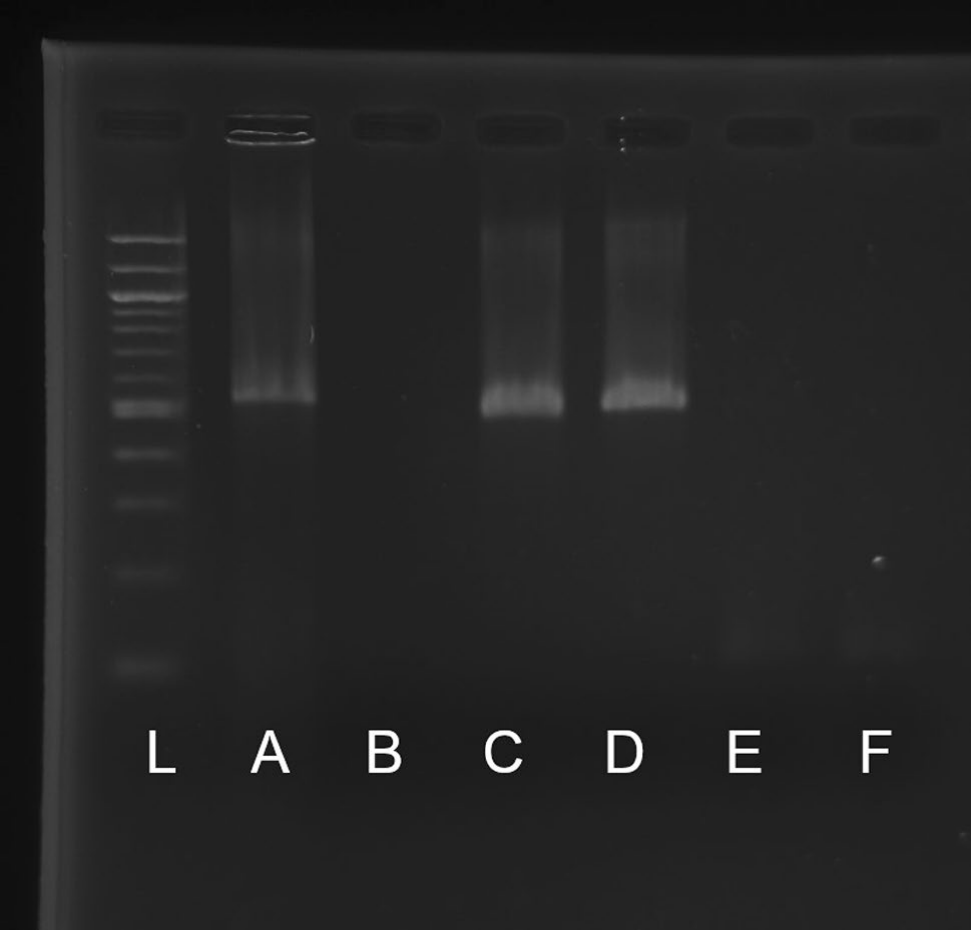
Molecular test of axenic condition. Gel electrophoresis (2% agarose) of 16S rRNA gene V3-V4 regions PCR amplification. (**L**) DNA marker 1000 bp (NEB) (**A**) positive control (bacterial culture, band at ~ 450 pb) (**B**) negative control (sterile water) (**C**) day 4 conventional condition (all replicate combined, band at ~ 450 pb) (**D**) day 4 axenic condition (all replicate combined) (**E**) day 20 axenic condition (all replicate combined) (**F**) negative control (ultrapure water).

Conventional larvae had higher enrichment in the digestive system, the lipid metabolism and carbohydrate metabolism pathways in early larvae (day 4) than larvae without microbiota (Fig. 7). Therefore, microbiota seems to have a positive influence on early larvae intake and transformation of nutrients into lipids.

In polyphagous insects, the major digestive enzymes carboxypeptidase A, carboxypeptidase B, aminopeptidase and the superfamily of serine endoproteinases (trypsin, trypsin-like enzyme, and chymotrypsins) play a major role in protein digestion and adsorption^72^. Larvae reared in axenic condition consistently had up-regulation of three trypsin (Trypsin eta, AC: P54629, trypsin II-P29, AC: Q90629, and trypsin-2, AC: P35032) at day 4, 12 and 20 (Table 1). Trypsin expression has been found to be stable in BSFL, even when kept in starvation state for an extended period of time^73^. Furthermore, some trypsins were up-regulated in conventional larvae, but not consistently across the three time points, and they never included the three persistently up-regulated trypsins found in the axenic larvae. This suggests that the microbiota’s presence induce a modulation of trypsin transcription in the host. Conventional larvae also had higher transcription for vasotab (AC: P84843), a vasodilator identified in the horse fly *Hybomitra bimaculata* after bloodmeal. To our knowledge, we are the first to provide evidence of an orthologous vasotab protein in *H. illucens*. This vasotab protein may play a role when BSFL feed on carcasses. The presence of blood components in the BHI media may have triggered its expression. GO annotation showed axenic larvae at day 4 and 12 had up-regulation of transmembrane transport activity for carbohydrates (alpha-glucoside, oligosaccharide, disaccharide, glucoside, sugar, and trehalose). Trehalose is the first resource used to produce energy in starvation conditions^74^. Starvation conditions lead to an increased mobilization of sugar and lipid nutrients from the fat body toward the hemolymph^75^. This supports the theory that axenic larvae were subjected to higher starvation stress than conventional larvae.

In low nutrient conditions, insects use their stored lipid resources by reduced glucose oxidation, combined with the mobilization of FA as well as lipid oxidation^57^. This was observed in axenic larvae at day 20, as its transcriptome profile found enrichment of *lipid catabolic process* GO.

The use of culture media also enabled us to observe the lack of molt residues left by larvae. *H. illucens* has been reported to molt up to six times during the larval cycle before entering the pre-pupal stage^76^. The lack of molt residues under axenic conditions may be due to (1) absence of molting or (2) digestion of molt residues by the larvae. The former hypothesis is dubious as the transcriptome profile at day 4 included the expression of the cuticle protein 6 (AC: P82119), reported to be expressed in post-ecdysial nymph (UniProt). As was previously reported^2^, BSFL produce degrading enzymes targeting cellulose and chitin. We were able to further confirm this, as the transcriptome profiles included lytic polysaccharide mono-oxygenase, cellulose-degrading enzyme (Pfam: PF03067.15), probable chitinase 2 (AC: Q9W02), probable chitinase 10 (AC: Q9W5U2), and chitinase-like protein Idgf4 (AC: QPQM7), this also supports the theory that BSFL consume their molt residues. This behaviour may have been exacerbated by the starvation stress; changes in behaviour are expected as adaptative responses to starvation in insects^57^.

### Growth and development

Conventional larvae had indications of greater neural development, such as the enrichment of the GO *peripheral nervous system development, sphingolipids and glycosphingolipids metabolic processes* at day 4 and 12. These GO were completely absent from the enrichment list associated with axenic larvae at any time point. Insect sphingolipids are essential to cellular homeostasis, developmental processes, differentiation, and neurogenesis^77^. Glycosphingolipids include gangliosides, responsible for neuronal differentiation and signaling in the nervous system^78^. We also found in four-days-old conventional larvae up-regulation of DETs encoding for ceramide phosphoethanolamine (AC: 077,475), believed to play an important role in early development of nervous system in *Drosophila*^79^. The higher rate of nervous system development in conventional larvae may have been stimulated by microorganisms’ presence, or been possible because, as we have previously established, conventional larvae had more resources to allocate towards nervous system development, or both.

Olfaction is a vital part of how insects interact with their environment. Previous studies revealed that through olfactive processes, the presence of microbiota affected the behaviour of oviposition in *H. illucens*^29^. We found that conventional larvae had greater olfaction related transcriptomic activity than larvae without microbiota at days 4 and 12.

## Conclusion

Our transcriptome analysis indicates that the microbiota modulates its host expression profile during ontogeny which suggests that the microbiota is essential to BSFL’s normal development. BSFL exempt of microbiota showed dampened transcriptomic activity in early (day 4) development associated pathways (*Aging, Cell growth and death, Endocrine system, Sensory system,* and *Signal transduction*), in digestive and nutrient intake pathways (*Carbohydrate metabolism, Lipid metabolism,* and *Transport and catabolism*) as well as in *Immune system* and *Xenobiotics biodegradation* pathways*.* Transcriptome expression was mostly affected in late larval stage (day 20) for nervous system, showing a long-term effect of microbiota on its host ontogeny.

This study revealed similarities as well as differences in the effect of microbiota in host transcriptome expression profile of the BSFL model compared to what was previously found in other insect models.

As the microbiota’s communities in BSFL are modulated by the diet on which the larvae are reared, it is essential to understand how the build-up of different microbial communities affect the host transcriptome profile along larval development. Such integrated understanding of larval development and performance opens the way for the development and optimization of various specialized industrial uses involving waste recycling and other applications.

## Data Availability

The raw datasets generated during the current study are freely available for non-commercial purposes in the NCBI Sequence Read Archive (SRA) public repository, accession: PRJNA814308 https://www.ncbi.nlm.nih.gov/bioproject/PRJNA814308.
