# Author Correction: Absence of microbiome triggers extensive changes in the transcriptional profile of *Hermetia illucens* during larval ontogeny

**DOI:** 10.1038/s41598-023-48023-6

**Published:** 2023-11-28

**Authors:** Laurence Auger, Sidki Bouslama, Marie-Hélène Deschamps, Grant Vandenberg, Nicolas Derome

**Affiliations:** 1 Département de Biologie, Université Laval, Quebec, QC Canada; 2 Institut de Biologie Intégrative et des Systèmes (IBIS), Département de Biologie, Université Laval, 1030 Avenue de la Médecine, G1V 0A6 Quebec, QC Canada; 3 Département des Sciences Animales, Université Laval, Quebec, QC Canada

Correction to: *Scientific Reports* https://doi.org/10.1038/s41598-023-29658-x, published online 10 February 2023

The original version of this Article contained an error, where the term “ontogeny” was incorrectly given as “ontology.”

As a result, in the title,

Absence of microbiome triggers extensive changes in the transcriptional profile of *Hermetia illucens* during larval ontology

now reads

Absence of microbiome triggers extensive changes in the transcriptional profile of *Hermetia illucens* during larval ontogeny

Also in the abstract:

These results provide the first evidence of host functional genes regulated by microbiota in the BSF larva, further demonstrating the importance of host-microbiota interactions on host ontology and health.

now reads:

These results provide the first evidence of host functional genes regulated by microbiota in the BSF larva, further demonstrating the importance of host-microbiota interactions on host ontogeny and health.

Also in the Introduction:

However, microbiota-brain axis during ontology has not been investigated to date.

now reads:

However, microbiota-brain axis during ontogeny has not been investigated to date.

Also in the discussion:

Here, we delved into how the microbiota’s presence (conventional condition) or absence (axenic condition) changes the transcriptome expression profile of the BSFL during larval ontology. This enabled us to gain insight into the most affected metabolic processes and biological pathways with subsequent functional annotation (UniProt), GO enrichment, and KEGG pathways enrichment. This study offers new insight into the host-microbiota interactions affecting BSFL ontology.

now reads:

Here, we delved into how the microbiota’s presence (conventional condition) or absence (axenic condition) changes the transcriptome expression profile of the BSFL during larval ontogeny. This enabled us to gain insight into the most affected metabolic processes and biological pathways with subsequent functional annotation (UniProt), GO enrichment, and KEGG pathways enrichment. This study offers new insight into the host-microbiota interactions affecting BSFL ontogeny.

and finally in the Conclusion:

Our transcriptome analysis indicates that the microbiota modulates its host expression profile during ontology which suggests that the microbiota is essential to BSFL’s normal development.

Transcriptome expression was mostly affected in late larval stage (day 20) for nervous system, showing a long-term effect of microbiota on its host ontology.

now reads:

Our transcriptome analysis indicates that the microbiota modulates its host expression profile during ontogeny which suggests that the microbiota is essential to BSFL’s normal development.

Transcriptome expression was mostly affected in late larval stage (day 20) for nervous system, showing a long-term effect of microbiota on its host ontogeny.

The original Article has been corrected.

